# Study of Quenching and Partitioning (Q&P) and Ultrasonic Surface Rolling (USR) Process on Microstructure and Mechanical Property of a High-Strength Martensitic Steel

**DOI:** 10.3390/ma17112752

**Published:** 2024-06-05

**Authors:** Yi Hou, Chenfeng Duan, Xiaoqiang Li, Shengguan Qu

**Affiliations:** 1 National Engineering Research Center of Near-Net-Shape Forming for Metallic Materials, Guangzhou 510640, China; yi_hou186@foxmail.com (Y.H.); 18636593866@163.com (C.D.); 2 School of Mechanical and Automotive Engineering, South China University of Technology, Guangzhou 510640, China; lixq@scut.edu.cn

**Keywords:** Q&P, bainite, USR, grain refinement, combination of strength and plasticity

## Abstract

Steel with a combination of strength and plasticity is prevalently demanded for lightweight design and emission reductions in manufacturing. In this study, a high-strength Cr-Ni-Mo martensitic steel treated by quenching and partitioning (Q&P) and ultrasonic surface rolling (USR) processes was studied for both strength and plasticity enhancement. Specimens were austenitized at 850 °C and then quenched to 240 °C via cooling by water, oil, and normalization in quenching. This was followed by partitioning, in which two groups of specimens were heated to 370 °C and 350 °C for 45 min, respectively. At last, all the specimens were quenched to room temperature with the same methods of quenching. The highest tensile strength increased from 681.73 MPa to 1389.76 MPa when compared to as-received (AR) steel after the Q&P process. The USR process with a static force of 800 N further improved the tensile strength of specimens with high tensile strength after the Q&P process, which improved from 1389.76 MPa to 1586.62 MPa and the product’s strength and elongation (PSE) increased from 15.76 GPa% to 15.9 GPa%, while the total elongation showed a mitigatory decrease from 11.34% to 10.02%. Tensile fractures were also studied and verified using a combination of strength and plasticity after a combined process of Q&P and USR.

## 1. Introduction

High-strength Cr-Ni-Mo martensitic steel is widely adopted in prop shafts and transmission shafts, etc., which have surfaces with high strength and cores with good plasticity, which are vital factors for dependability, efficiency, and emission reductions. This characteristic promises wear and fatigue resistance with high torsion strength, especially for lightweight-designed components operating at high power density [1,2,3]. The lightweight-designed component has been greatly supported by heat treatment and other surface-strengthening methods such as ultrasonic surface rolling (USR) and shot peening, which is applied in industries concerning vehicles, aerospace, shipping, etc., for energy conservation [4,5,6].

The different heat treatment parameters of Cr-Ni-Mo steel have a great influence on microstructure, which could induce an obvious mechanical property difference [7]. Bainite induced by the quenching and partitioning (Q&P) process is a promising method for both strength and plasticity enhancement, which is induced from austenite in quenching. In partitioning, bainite disturbs the carbon transformation from martensite to austenite, and a gradient carbon distribution is formed [8]. This process induces a multitude of filmy retained austenite (RA) close to bainite with a high carbon concentration and good stability, while the area separate from bainite has more pronounced carbon precipitation, and blocky RA in a low carbon concentration is formed with less stability. Blocky RA and martensite form martensite/austenite (M/A) islands, which are split into smaller grains by bainite [9,10,11]. A high content of stable RA promotes the transformation-induced plasticity (TRIP) effect when steel is deformed by an external force, in which RA would transform into martensite with phase transformation strengthening and plastic growth as cracks nucleate and cluster around martensite. An increased content of martensite leads to enhanced local hardness, which causes cracks to propagate in its surroundings and delayed necking. As a result, the strength and plasticity of steel are enhanced simultaneously [12,13]. Also, the addition of other elements could affect the formation of bainite and the mechanical properties of steel. Liu et al. studied stepped partitioning (SP) to enhance the yield strength (YS) of medium-carbon silicon-rich steel. The SP process with an extended period guaranteed a high content of small carbides in martensite, thus contributing to an increase in YS. In addition, SP achieved a stable RA for tensile strength and ductility improvement [14]. Dai et al. simulated the extension of bainitic ferrite in an Fe-C alloy steel to study the excess of carbon and extension rate for bainitic formation. It was demonstrated that temperature had a great influence on excess carbon in bainitic ferrite, which induced a carbon solute drag effect, reducing the carbon excess and fettering the growth of bainitic ferrite [15]. Liu et al. studied the product of strength and elongation (PSE) variation with the amount of isothermal martensite and bainite under different Q&P processes. Quenching time had a positive effect on the mechanical properties of steel via an increase in isothermal martensite. Bainite also had an influence on the PSE, in which a partitioning temperature lower than bainite’s starting temperature could induce bainite, and more bainite was obtained by decreasing the partitioning temperature for a more stable RA and higher PSE [16].

As a flexible and efficacious method, the USR process could further enhance the strength of steel via surface strengthening [17]. The USR process induces severe plastic deformation on the steel surface with high pressure, which refines grains and induces a hardened layer on the steel. Refined grains have more grain boundaries, and RA in the hardened layer is transformed into martensite for local hardness enhancement due to plastic deformation. With this function, the USR process has a notable function of restraining crack propagation, and the tensile strength of steel is significantly enhanced [18,19,20]. Also, severe plastic deformation induces a significant reduction in surface roughness and a sharp increase in surface hardness [21]. Qin et al. researched the influence of the USR process on the tensile performance of quenching–partitioning–tempering-treated steel. The hardened layer helped to avoid strain localization and induced a biaxial stress state. In addition, the twinned martensite and refined grain size also contributed to the performance increase [22]. Xu et al. investigated the mechanical properties of FH36 marine steel at a low temperature after the USR process. The grain size was reduced from 10.92 μm to 5.86 μm and a nanostructured surface layer was induced. The surface roughness decreased notably with Ra under 0.074 μm, and the hardened layer had a depth of 580 μm. The yield strength and ultimate tensile strength were greatly enhanced after USR treatment, which was promoted by severe plastic deformation in the USR process [23].

High-strength Cr-Ni-Mo martensitic steel has been greatly studied in surface strengthening, and the strength could be further improved by the Q&P process for superior plasticity in the core, while the surface strength is enhanced by the USR process to improve the mechanical properties of steel. In this paper, the combined process of Q&P and USR was studied to investigate both strength and plasticity enhancements. The Q&P process optimizes both strength and plasticity via bainite and filmy RA. The USR process further enhances strength via severe plastic deformation and refined grain size. The microstructure, volume fraction of RA, tensile strength, total elongation, and PSE were compared before and after the Q&P process under different quenching and partitioning conditions. The specimens with high tensile strength after the Q&P process were further treated by the USR process for the surface-strengthening study. Tensile fractures were also analyzed to demonstrate the influence of microstructural evolution and surface strengthening on mechanical property enhancement.

## 2. Experimental Procedures

The Cr-Ni-Mo steel applied in this paper was obtained via spheroidal annealing, continuous rolling, and the fine tuning of raw material, and compositions are listed in Table 1.

**Figure 1:**
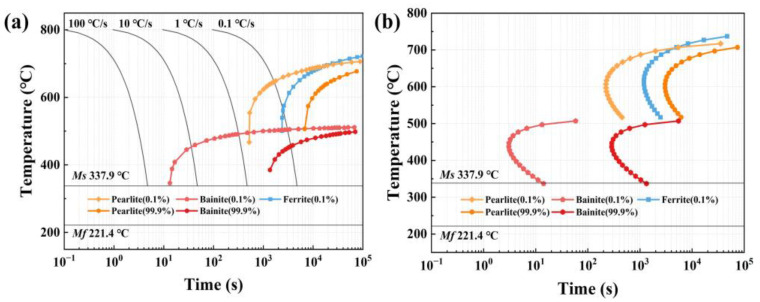
(**a**) CCT and (**b**) TTT curves of Cr-Ni-Mo steel.

The continuous cooling transformation (CCT) curve and time–temperature transformation (TTT) curve were simulated with JmatPro (Public release version 7.0.0, Sente Software, Guildford, UK) and are shown in Figure 1 to verify the heat treatment characteristics of steel. As the results indicate, *Ac*_3_ of steel is 812.2 °C, *Ms* is 337.9 °C, and *Mf* is 221.4 °C. From Figure 1, bainite could be induced above *Ms*, and it is precipitated before pearlite and ferrite with a lower precipitation temperature. The *Ac*_3_ and *Ms* of steel were double-checked using an empirical equation based on the compositions in Table 1 as [24]:

(1) $${Ac}_{3}\left({}^{\circ}C\right)=910-203\sqrt{C}-15.2Ni+44.7Si+104V+31.5Mo$$

(2) $$Ms\left({}^{\circ}C\right)=512-453C-30.4Mn-16.9Ni+15Cr-9.5Mo+{217C}^{2}-71.5C\cdot Mn-67.6C\cdot Cr$$

and the results from Equations (1) and (2) are 807.0 °C and 334.2 °C, respectively, which demonstrates the correctness of the simulation results.

**Figure 2:**
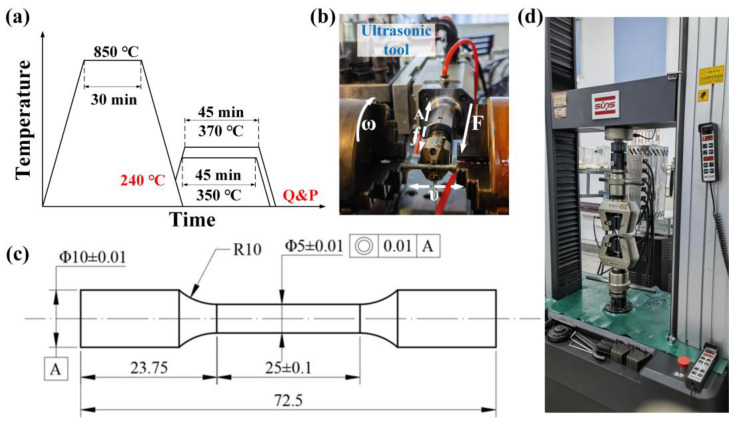
Schematic image of (**a**) the Q&P process, (**b**) the USR process, (**c**) specimen dimensions with unit in mm, and (**d**) the tensile test.

According to simulation results, the Q&P process was designed as in Figure 2a and conducted with a resistance furnace (KSL-1200X-M, Hefeikejing, Hefei, China). Specimens were austenitized at 850 °C for 30 min and then quenched to 240 °C by water, oil, and normalization. In partitioning, two groups of specimens were heated to 370 °C and 350 °C for 45 min, respectively. At last, all the specimens were quenched to room temperature with the same quenching methods from 850 °C to 240 °C. The cooling speed of specimens in water was approximately 30 deg/s and in oil was approximately 10 deg/s, and for specimens undergoing normalization, was approximately 0.3 deg/s [25]. The duration of specimen quenching from 850 °C to 240 °C in water was approximately 20 s, approximately 61 s in oil, and approximately 2033 s for normalizing. Quenching time from partitioning temperatures to room temperature was more than 12 h for total cooling. To further study the mechanical property enhancement after the USR process, specimens with high performances after the Q&P process were treated by the USR process. The control groups listed in Table 2 were compared to study the influence of the quenching method, the partitioning temperature, and the USR process on the microstructural evolution and mechanical properties of high-strength Cr-Ni-Mo martensitic steel.

Figure 2b shows the USR process, that the tool of ultrasonic strengthening system (HKN540, Huayun, Jinan, China) was mounted on a lathe to feed forward and backward in tempo υ. Static force F, ultrasonic vibration with amplitude A, and the frequency were generated by the system and transmitted to the tool. The carbide alloy ball on the tip of the ultrasonic tool was in contact with the specimen and rolled with the feed of the ultrasonic tool as a chuck rotating in palstance ω. Simultaneously, static force F was converted into high pressure, together with high-frequency vibration inducing severe plastic deformation on the specimen’s surface. This process refines the grain size for surface strengthening. In addition, lubricant flowed abidingly between the ball and the specimen surface to avoid heat accumulation and sliding. Each specimen was treated four times, and three specimens were treated by the USR process for Q&P370 WU and Q&P350 NU, respectively. The specific parameters applied in the USR process are listed in Table 3. The dimensions of specimens applied in this paper for the Q&P process, the USR process, and the tensile test are shown in Figure 2c.

A scanning electron microscope system (SEM; Quanta 200, FEI, Hillsboro, OR, USA) with an energy dispersive spectrometer (EDS) was utilized for microstructural morphological observation and element qualitative analysis. The optical microscope (OM; DMi8 C, Leica Microsystems CMS GmbH, Mannheim, Germany) was used for the USR hardened layer observation. The phase change and calculation of the volume fraction of RA were conducted with X-ray diffraction (XRD; smartlab, Rigaku, Tokyo, Japan) under Cu-Kα excitation radiation (λ = 1.54056Å) with a 2θ range of 30–100° and step of 0.01°. The surface profile of the specimen before and after the USR process was measured with a surface profiler (MarSurf M300C, Mahr, Esslingen, Germany), and the hardness of each specimen was measured three times using a Vickers tester (DHV-1000Z, SCTMC, Shanghai, China) under 1.96 N (200 gf) and 15 s. The tensile test was performed on a universal testing machine (UTM5105, SUNS, Shenzhen, China) as shown in Figure 2d, and the results are shown as the average values of all three tests for each variable.

## 3. Results and Discussion

### 3.1. Q&P Process

#### 3.1.1. Microstructure Evolution

**Figure 3:**
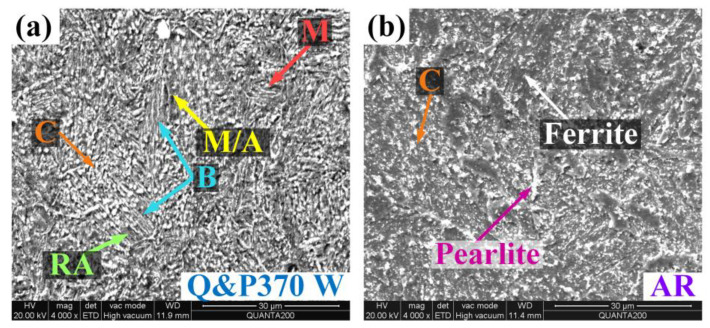
Microstructural morphology of (**a**) Q&P370 W and (**b**) AR; AR: as-received specimen, B: bainite, C: carbide precipitate, M: martensite, M/A: martensite/austenite (M/A) islands, RA: retained austenite, W: water.

Figure 3 shows the microstructural morphology of the specimens under different Q&P processes and with AR specimens. For Q&P370 W in Figure 3a, large areas of dense acicular bainite appeared after the Q&P process, which were quenched by water, and granular carbide precipitate was distributed around the bainite. A clear distribution of blocky RA can be observed in the regions separate from bainite with a low concentration of carbide precipitate, and blocky RA is broken into fine particles. M/A islands composed of carbon-depleted austenite and martensite are found around blocky RA, which are also split by bainite. Lath martensite is short and thick in shape and is split by bainite and carbide precipitates that are interspersed with fewer carbide precipitates in the location separate from bainite. This morphology also shows a gradient carbon distribution. In comparison, the microstructure morphology of AR in Figure 3b is composed of prominent rough ferrite and pearlite in coarse nubs, and small carbide particles are spread between ferrite and pearlite.

In the Q&P process, specimens were first quenched from 850 °C to 240 °C as a quenching temperature between *Ms* and *Mf* could induce the phase transformation from austenite to bainite and martensite. With the appearance of bainite, RA, M/A islands, and martensite were split into smaller sizes. Then, in partitioning, the temperature was increased for carbon redistribution among bainite, martensite, and RA. In this process, the types and parameters of RA such as the size, volume, stability, and location were also determined, which has a great influence on the mechanical properties of steel [26]. Due to the addition of Si, bainite appeared preferentially in low-carbon content areas and increased the carbon content of adjacent RA areas, but Ni had a negative influence on the bainite transformation [27,28]. The appearance of bainite-enriched filmy RA inside or between bainite with high-carbon concentrations. Filmy RA is much more stable due to the higher carbon content, and the TRIP effect can be guaranteed with a high content of stable RA for better mechanical properties. In comparison, blocky RA with low-carbon concentrations is less stable and brittle, which has a negative effect on the steel’s performance and induces M/A islands [29]. In Figure 3a, fewer carbide precipitates are entrained in bainite and more carbide is found around RA and M/A islands, and martensite also contains small amounts of carbides. This gradient carbon distribution proves a high carbon absorption in filmy RA, and blocky RA has less dissolved carbon.

**Figure 4:**
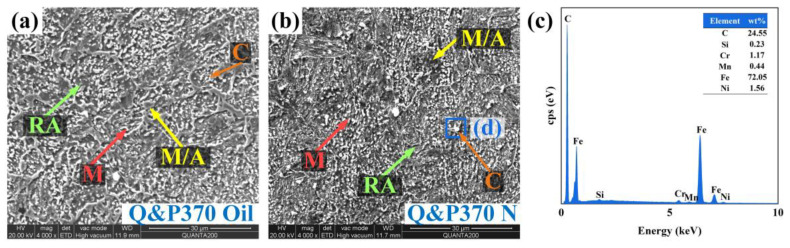
Microstructural morphology of (**a**) Q&P370 Oil, (**b**) Q&P370 N, and (**c**) elementary analysis of area d in (**b**), C: carbide precipitate, M: martensite, M/A: martensite/austenite (M/A) islands, N: normalizing, RA: retained austenite.

Figure 4a,b shows specimens partitioned at 370 °C and quenched by oil and normalizing, respectively. Oil has a faster cooling speed than normalizing but is slower than water, while both Q&P370 Oil and N have no bainite induced. Cooling speed has a notable influence on microstructural evolution, and the formation of bainite in an acicular shape appears in the proper speed range [30]. In Figure 4a, the component is mainly coarse M/A islands with blocky RA. Carbide precipitates with small sizes are uniform among M/A islands, RA, and lath martensite. This morphology proves that most carbon was not absorbed by RA or martensite but was rather precipitated. As the cooling speed decreased further, the proportion of RA significantly increased, as shown in Figure 4b. Blocky RA segregates M/A islands and induces lath martensite in short and thick shapes. An uneven distribution of carbide could be observed, and the elementary analysis is shown in Figure 4c. The gradient distribution of carbon in partitioning instead of bainite determines the microstructure morphology and mechanical properties of the specimen as the cooling speed decreased; also, the type of RA varied from Q&P370 W [31]. The carbon content of austenite determines the stability of RA, and a higher content shows a better TRIP effect and enhances the performance of steel. In addition, carbon homogenization in RA takes a long time, as carbon spreads gradually from the edge to the interior of RA. Smaller blocky RA could speed up this process, together with a slower cooling speed, as normalizing allows full carbon diffusion and uniform distribution in blocky RA. Also, smaller blocky RA refines the size of M/A islands and martensite, and better performance of steel could be guaranteed [32,33]. In comparison, Q&P370 Oil is mainly composed of coarse M/A islands, which proves that a short carbon diffusion time induces a rough component size and low carbon diffusion, leading to poor mechanical properties of the steel.

**Figure 5:**
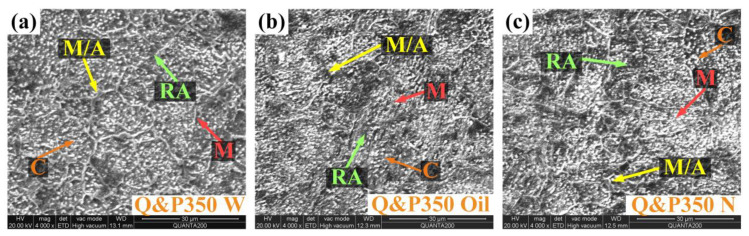
Microstructural morphology of (**a**) Q&P350 W, (**b**) Q&P350 Oil, and (**c**) Q&P350 N, C: carbide precipitate, M: martensite, M/A: martensite/austenite (M/A) islands, N: normalizing, RA: retained austenite, W: water.

As the partitioning temperature decreases to 350 °C, no bainite appears and the grain is generally coarser than under 370 °C. This could be explained by the tendency of thermodynamic equilibrium transformation in bainite, which influences the content of bainite [34]. In addition, a lower partitioning temperature leads to a greater driving force for bainite transformation, but the function of atomic diffusion is weak. As a result, bainite transformation in a low partitioning temperature is restrained especially when it has the same transformation time as Q&P370 [27]. Also, a lower partitioning temperature causes a mild carbon redistribution, and as shown in Figure 5a–c, a slower cooling speed leads to more time for carbon redistribution. The size of blocky RA is reduced with a slower cooling speed [35]. Rough blocky RA is the main component in the Q&P350 process, and the stability of RA is related to the uniformity of the carbon distribution. The location and size of martensite are also determined by the location and concentration of carbide precipitates [36]. The distribution of carbide precipitates in Figure 5a is even, while carbon distribution is less uniform in Figure 5b,c, which demonstrates an adequate carbon redistribution process and improvement in the stability of blocky RA. The cooling speed has a notable influence on the carbon distribution. In Figure 5c, the carbon distribution is more aggregated, and blocky RA is separated into small pieces; also, the component size is further reduced via quenching in water and oil [37].

The carbon content of blocky RA was also measured by EDS, and the results are listed in Table 4. The result has the same tendency as that analyzed above, in which bainite could disturb the carbon distribution in partitioning and cause a low carbon content in blocky RA in Q&P370 W. For Q&P 370 Oil and N, a slower cooling speed of normalizing guarantees full carbon diffusion, and the carbon content of blocky RA is higher than it is in Q&P 370 Oil. As the cooling speed slows down from water to normalizing in Q&P350, a mild carbon redistribution promises a higher carbon content in blocky RA.

#### 3.1.2. XRD and Volume Fraction of RA

**Figure 6:**
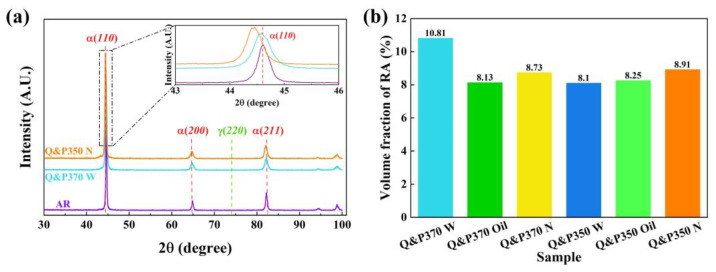
The results of (**a**) XRD and (**b**) volume fraction of RA under different Q&P processes.

The XRD results of the specimens after Q&P370 W and Q&P350 N are shown in Figure 6a and compared with AR. The peaks of the three specimens have a notable difference in α(110). The peaks of Q&P370 W and Q&P350 N shifted to lower angles, and Q&P370 N had a more pronounced shifting tendency. These results indicate that the martensite of those two specimens was filled with carbon and expanded. More carbon is observed in the martensite of Q&P350 N, leading to a coarser grain size [38]. Also, more carbon in martensite causes a lower carbon concentration in RA, and RA in Q&P350 N is less stable.

The volume fraction of RA in Figure 6b is calculated based on the peak intensity of α(211) and γ(220) as [28]:

(3) $$V_{RA}=\frac{1}{1+0.65\cdot \frac{l_{1}}{l_{2}}}\times 100\%$$

where $l_{1}$ is the peak intensity of α(211) and $l_{2}$ represents the peak intensity of γ(220). Q&P370 W has the highest volume of RA at 10.81%, in which the induction of bainite promotes the transformation of filmy RA with high stability and a limited amount of blocky RA with fewer carbide precipitates located around bainite. Q&P370 Oil has the lowest RA volume of 8.13%. Also, less RA is observed in Figure 4a, proving that coarse M/A islands constitute the main component of the specimen. The RA volume of Q&P370 N increased to 8.73% with blocky RA with high carbon content and high stability. The smaller sizes of RA, M/A islands, and martensite in Figure 4b could be explained by the fact that the full dissolution of carbon refines the size of blocky RA and splits M/A islands and martensite into smaller sizes [39]. For specimens treated by the Q&P350 process, the RA volume exhibits a limited difference among different quenching methods and is much lower than it is in Q&P370 W. The RA volumes of Q&P350 W, Oil, and N are 8.1%, 8.25%, and 8.91%, respectively. This result proves that carbon redistribution during the Q&P350 process is mild, and the absence of bainite reduces the stability of RA. The adequate redistribution of carbon induces blocky RA, and a lower cooling speed promotes a higher RA volume.

#### 3.1.3. Mechanical Property

**Figure 7:**
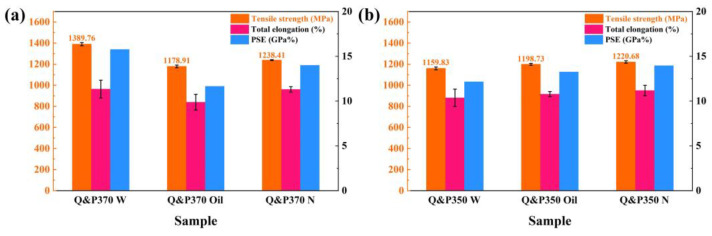
The tensile test results of (**a**) Q&P370 and (**b**) Q&P350 processes.

Figure 7 exhibits the results of tensile strength, total elongation, and PSE of specimens treated by Q&P370 and 350 in different cooling methods. In Figure 7a, Q&P370 W has the highest tensile strength of 1389.76 MPa and a total elongation of 11.34%. This is followed by Q&P370 N in 1238.41 MPa and 11.3%. Q&P370 Oil has the worst mechanical properties of 1178.91 MPa and 9.88%. For Q&P370, PSE has the same tendency as total elongation with 15.76 GPa%, 11.65 GPa %, and 13.99 GPa% for W, Oil, and N, respectively. It is demonstrated that stable filmy RA together with the refined component size in Q&P370 W exhibits superior mechanical properties. As analyzed above, the TRIP effect is determined by the type, size, volume, and stability of RA, which is vital to the mechanical properties of steel. Hence, steel treated by the Q&P370 W process contains a high volume of filmy RA and exhibits a combination of strength and plasticity as superior mechanical properties compared to oil and normalizing. Q&P370 N has a rich content of small blocky RA with high stability and refined M/A islands and martensite. The smaller component size with good plasticity in Q&P370 N retards crack propagation, and the total elongation is close to that of Q&P370 W, but the specimen with no filmy RA shows a noticeable decrease in tensile strength [40]. Q&P370 Oil performs the worst in tensile strength due to the coarse component size and low stability of blocky RA; also, the high content of brittle M/A islands and martensite leads to poor total elongation.

As shown in Figure 7b, specimens with mild carbon redistribution lead to lower tensile strength, and a longer carbon diffusion time induces a relatively more stable blocky RA and a reduced RA size. Tensile strength is also increased as the cooling speed slows down from water to normalizing. Q&P350 W has a tensile strength of 1159.83 MPa, which could be explained by the even distribution of carbide precipitates [41]. As the cooling speed slows down, Q&P350 Oil has a higher tensile strength of 1198.73 MPa, and Q&P350 N has the highest at 1220.68 MPa. Blocky RA in Q&P350 has less stability than filmy RA in Q&P370 W. Although sufficient carbon dissolution reduces the size of blocky RA, the size of blocky RA in Q&P350 N is still greater than in Q&P370 W with no partitioning effect to form bainite. At the same time, as the XRD results indicate, martensite is filled with carbon with rough sizes, which causes a much lower tensile strength than Q&P370 W. Blocky RA and the coarse size of Q&P350 result in an insignificant increase in the total elongation, which is 10.36%, 10.78%, and 11.18% from water to normalizing. PSE also has a similar tendency to increase with values of 12.15, 13.25, and 13.65 GPa% for Q&P350 W, Oil, and N, respectively [42]. Q&P370 W and Q&P350 N have the best mechanical properties in Q&P370 and Q&P350, respectively; thus, Q&P370 W and Q&P350 N were studied further to analyze the variation in hardness, XRD, and tensile strength after the USR process.

### 3.2. Combined Process of Q&P and USR

#### 3.2.1. Surface and Hardness Variation

**Figure 8:**
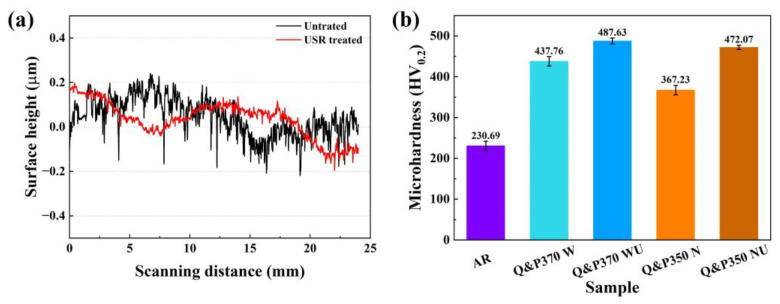
(**a**) Surface profile of AR, Q&P370 WU, and Q&P350 NU, and (**b**) surface hardness comparison of Q&P and USR processes; U: USR-treated.

The surface heights of specimens before and after the USR process are shown in Figure 8a. AR has a rough surface. After the USR process, the surface height was notably reduced. Due to severe plastic deformation, rough projection on the surface was pressed by high pressure and ultrasonic vibration and imposed into surface grooves. The pressed part was then re-bonded with grooves, and the surface roughness was reduced. This process induced a hardened layer, and a comparison of the surface hardness values is listed in Figure 8b. The AR specimen had the lowest value with 230.69 HV_0.2_ for the rough component. Specimens treated by the Q&P process showed a notable hardness increase, but different microstructural evolution induced hardness variation. With component refinement induced by bainite, Q&P370 W showed a noticeable hardness increase to 437.76 HV_0.2_ and further improved to 487.63 HV_0.2_ after the USR process. Q&P350 N showed a limited hardness improvement to 367.23 HV_0.2_, as the coarse blocky RA and martensite in Q&P350 N had a negative influence on hardness. The hardness difference between Q&P370 W and Q&P350 N could also be validated by the fact that, for Q&P350 N, more carbon is observed in martensite, leading to a coarser component size. However, a notable hardness increase in Q&P350 NU was obtained, which is further analyzed by gradient hardness.

**Figure 9:**
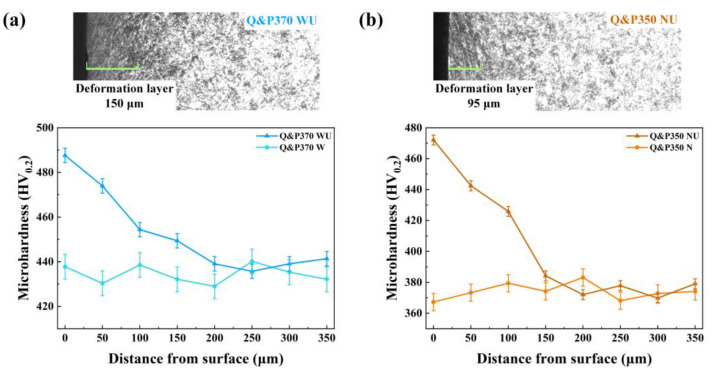
OM images and gradient hardness of (**a**) Q&P370 WU and (**b**) Q&P350 NU.

Gradient hardness was measured to explore the hardness distribution and depth of the hardened layers of Q&P370 WU and Q&P350 NU, as shown in Figure 9. Q&P370 WU has a deeper deformation layer than Q&P350 NU because for Q&P370 WU, a high content of filmy RA promotes superior plasticity. In Figure 9a, the grain size near the surface was significantly reduced due to severe plastic deformation. Also, a deep hardened layer with a slowly decreasing hardness gradient distribution was induced after the USR process. Bainite in Q&P370 W splits blocky RA, martensite, and M/A islands into smaller sizes, which guarantees the high hardness of steel. After the USR process, grain size refinement on the surface leads to an increase in surface hardness, and the structural properties of a hard surface and high plasticity in the core promote the mechanical properties of the combination of strength and plasticity. In comparison, the depth of the hardened layer in Q&P350 NU is shallow with a dramatically reduced hardness gradient. For Q&P350 N, blocky RA and martensite are the main components of the specimen, which induces hard and brittle characteristics. In Figure 9b, the grain size near the surface is smashed into smaller sizes, which enhances the surface hardness compared to before the USR process. However, the depth of the hardened layer is impeded by blocky RA and martensite, and the deformation layer is shallow. As a result, the USR process causes a more hardened surface with a limited depth of the hardened layer, and the hardness gradient is significantly reduced. In addition, the microstructure of Q&P350 NU below the hardened layer is still hard and brittle with coarse blocky RA and martensite, and the mechanical properties of Q&P350 NU are much more brittle. The results prove that the USR process has a superior effect on steel with high plasticity, and the combination of strength and plasticity can be achieved using surface strengthening [43].

#### 3.2.2. XRD Analysis

**Figure 10:**
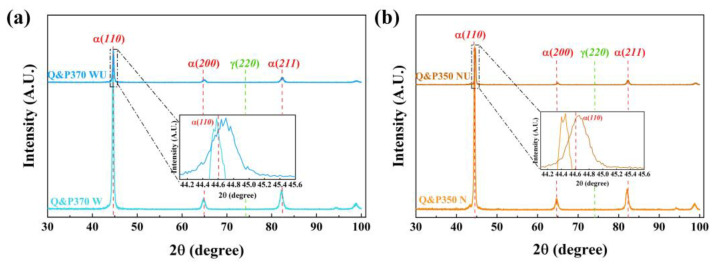
XRD comparison of (**a**) Q&P370 W and Q&P370 WU, and (**b**) Q&P350 N and Q&P350 NU.

Figure 10 exhibits the XRD results before and after the USR process of Q&P370 W and Q&P350 N. Both α(110) peaks of the two specimens are shifted to the right, indicating the appearance of a residual compressive stress field after severe plastic deformation in the USR process. A greater peak-shifting tendency of Q&P370 WU implies that the plastic deformation in Q&P370 WU is more severe [44]. This result is consistent with the analysis above that Q&P370 W has high plasticity, the USR process could induce a deeper hardened layer, and plastic deformation is more adequate after the USR process. In comparison, Q&P350 N is more brittle, which prevents the deformation layer from extending to the core in the USR process and causes a faint peak-shifting tendency in Q&P350 NU [45]. The full width at half maximum (FWHM) could indicate the grain size variation, in which a wider FWHM indicates a smaller grain size. The FWHM values of the two specimens are wider than those before the USR process, proving notable grain refinement after the USR process [46]. For Q&P370 W, the specimen is harder and more plastic than Q&P350 N, and hard grains are more easily refined and a deeper plastic deformation layer induces more refined grains in Q&P370 WU. The grains in Q&P350 N are more brittle with a high content of RA, which is also easily smashed into much smaller sizes. However, the brittleness of Q&P350 N had a negative effect on the hardened layer depth, thus fewer grains were refined in Q&P350 NU.

#### 3.2.3. Mechanical Property

**Figure 11:**
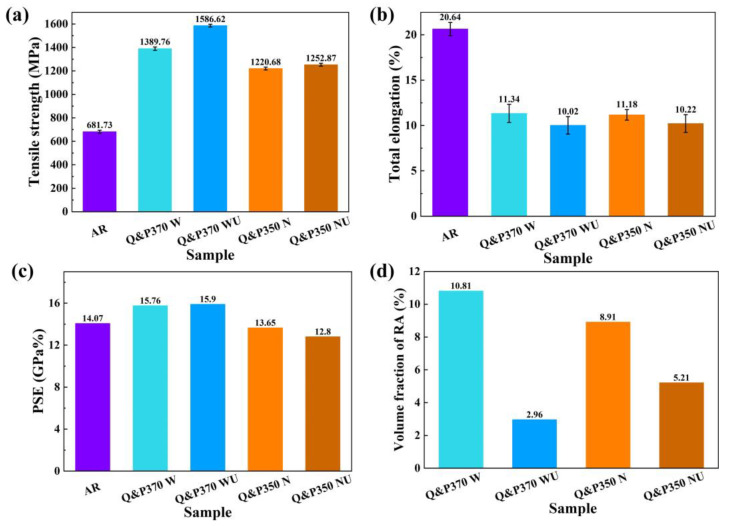
Tensile test results of (**a**) tensile strength, (**b**) total elongation, (**c**) PSE, and (**d**) volume fraction of RA for AR and specimens of Q&P370 W and Q&P350 N before and after the USR process.

The tensile test results of specimens treated by a combined process of Q&P and the USR are compared with Q&P-treated specimens and AR specimens and the results are listed in Figure 11 as the tensile strength, total elongation, PSE, and volume fraction of RA. In Figure 11a, Q&P370 WU has the highest tensile strength of 1586.62 MPa and showed an increase of 14.17% compared to Q&P370 W in 1389.76 MPa, while the tensile strength of AR is 681.73 MPa. The Q&P process significantly improves the strength of steel, and the USR process induces a further enhancement in strength with a deep hardened layer. In comparison, the tensile strength of Q&P350 N is 1220.68 MPa, and it improved to a limited extension of 1252.87 MPa after the USR process due to the low plasticity of Q&P350 NU. A more pronounced strength increase could be obtained by the USR process when steel has better plasticity, and the combination of strength and plasticity could be achieved. In comparison, the limited strength increase in Q&P350 N demonstrates that a hard and brittle surface hinders the formation of a hardened layer in the USR process, thus having a negative influence on strength improvement. The tensile strength of the AR specimen also illustrates that steel with a rough component size is poor in tensile strength, thus showing the effect of mechanical performance enhancement by the combined process of Q&P and USR process.

For total elongation in Figure 11b, AR has the highest value of 20.64%, and the Q&P process reduces total elongation to 11.34% and 11.18% for Q&P370 W and Q&P350 N, respectively. The USR process further decreases the total elongation to 10.02% for Q&P370 WU and 10.22% for Q&P350 NU. The coarse component size in AR indicates high plasticity but low strength. The decrease in total elongation leads to a significant increase in strength, but the Q&P process with USR treatment induces a different mechanism for a total elongation decrease. For Q&P370 W, stable filmy RA induces the TRIP effect to postpone the nucleating and clustering of cracks. The USR process inflicts a deep hardened layer and enhances the strength of the specimen, and the hardened layer reduces the surface plasticity and the total elongation decreases by 11.64%. In comparison, specimens treated by Q&P350 N are composed of unstable blocky RA and brittle martensite with coarse sizes, and total elongation also noticeably decreases from AR and is lower than Q&P370 W, which indicates the characteristic of brittleness. In the USR process, the hard microstructure of Q&P350 N induces a shallow hardened layer, leading to a lower total reduction in elongation in Q&P350 NU [47].

In Figure 11c, Q&P370 WU has a PSE of 15.9 GPa% and Q&P370 W is 15.76 GPa%, while the PSE of Q&P350 NU and Q&P350 N is 12.8 GPa% and 13.65 GPa%, respectively. For Q&P370 WU, a high increase in tensile strength compensates for the total elongation decrease and exhibits a combination of strength and plasticity. In addition, the specimen of Q&P370 WU has a hard surface, a deep hardened layer, and a plastic core after the USR process, which is vital for the high performance of components in a lightweight-designed structure. However, it should be noted that the depth of the hardened layer is critical to the performance enhancement of steel. A shallow layer could only provide a limited strength effect, but a deep layer also induces a negative influence on the plasticity of steel and even worsens the comprehensive mechanical property. It can be illustrated by Q&P370 WU that although a deep hardened layer significantly promotes tensile strength when compared to Q&P370 W, it also notably reduces total elongation. For the shallow hardened layer in Q&P350 NU as in Figure 9b, the brittle specimen has a limited effect on the surface strength and tensile strength, but the decrease in total elongation is also milder than in Q&P370 WU and Q&P370 W. This result indicates that the characteristics of steel should be considered before the USR process, and the proper depth of the hardened layer could achieve the comprehensive enhancement of mechanical properties for both strength and plasticity.

The volume fraction of RA is calculated using Equation (3) and compared in Figure 11d. Q&P370 WU has an RA volume of 2.96% while Q&P370 W is 10.81%. The RA volume of Q&350 NU is 5.21%, and Q&P350 N is 8.91%. Specimens treated by the USR process exhibit an obvious decrease in RA volume and prove the phase transformation in the USR process. This result is consistent with hardened layer depth analysis under different Q&P processes. Q&P370 WU has a deeper hardened layer and more filmy RA is transformed into martensite, causing a significant reduction in the RA volume of 72.62% from Q&P370 W to Q&P370 WU. The shallow hardened layer depth in Q&350 NU could only provide a limited amount of blocky RA for martensite transformation, in spite of the instability of blocky RA that makes the RA transformation easier to induce. The RA volume of Q&350 NU decreased by 41.53% compared to Q&350 N. The filmy RA transformed in Q&P370 WU indicates a deep hardened layer, while the remaining filmy RA in the core with high plasticity induces the TRIP effect and promotes plasticity. In comparison, a limited amount of blocky RA is transformed in the shallow hardened layer in Q&P350 NU, and unstable brittle blocky RA in the core has less function in the improvement of tensile strength, resulting in a minute tensile strength variation of Q&P350 N before and after the USR process.

**Figure 12:**
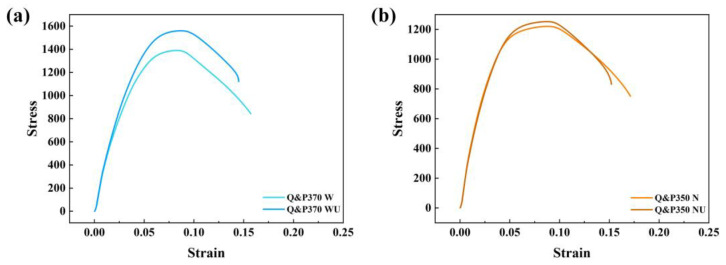
Strain–stress curve of (**a**) Q&P370 W, WU and (**b**) Q&P350 N, NU.

Figure 12a,b shows the strain–stress curve of Q&P370 W, WU and Q&P350 N, NU, respectively. The results have the same tendency as tensile stress and total elongation, in which specimens have higher tensile strength but a lower total elongation after the USR process than before the USR process. As analyzed above, the hardened layer induces a higher surface strength but negatively affects the plasticity of the specimen. Also, Q&P370 W, with its superior plasticity, induces a deeper hardened layer, and the variation in tensile stress and total elongation is more significant than that of Q&P350 N and NU in higher brittleness.

### 3.3. Tensile Fracture Analysis

#### 3.3.1. Fracture of AR

**Figure 13:**
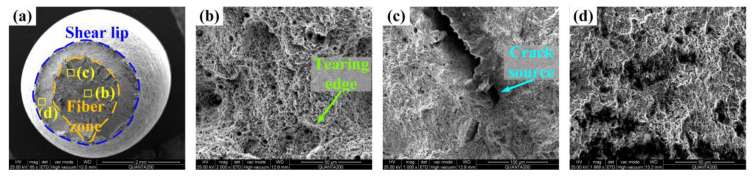
Tensile fracture of AR. (**a**) full view, (**b**) tearing edge in fiber zone, (**c**) crack source in fiber zone, and (**d**) shear lip.

Figure 13 shows the fracture of AR. In Figure 13a, the shear lip occupies a large section area, and the fiber zone is irregularly rounded, indicating an obvious plastic fracture process. Also, significant undulations in the fiber zone prove the difficulty in crack expansion as a sign of high plasticity [20]. In Figure 13b, large dimples are surrounded by tiny dimples that are densely distributed in the core, together with the tearing edge in bright white, indicating that a large amount of energy is absorbed in the fiber zone in the fracture process. In Figure 13c, fewer dimples are observed around the crack source, and the shear lip shown in Figure 13d has the characteristic of a cleavage fracture. The fracturing of AR demonstrates that the coarse component size exhibits plasticity in the microstructure fracture morphology, especially in the fiber zone, and induces an obvious cleavage fracture in the shear lip in the instantaneous fracture.

#### 3.3.2. Fracture of Q&P370 W and WU

**Figure 14:**
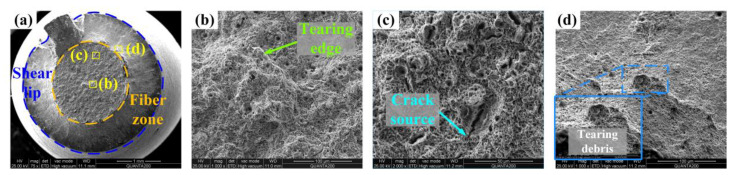
Tensile fracture of Q&P370 W. (**a**) full view, (**b**) tearing edge in fiber zone, (**c**) crack source in fiber zone, and (**d**) shear lip.

The fracture of Q&P370 W shown in Figure 14a has a regular round fiber zone, and the shear lip takes up more section area than in the AR fracture. Also, undulations in the fiber zone are not significant. This fracture morphology indicates lower plasticity of the specimen than AR, while tensile strength shows an obvious enhancement, as analyzed above. In Figure 14b, dimples with obvious size differences are evenly distributed with a smaller tearing edge, indicating good plasticity in the core. The crack extension is well restrained as no long cracks are observed in the tensile fracture. In Figure 14c, the crack is surrounded by a large number of dimples [48]. Cracks originate in M/A islands and when extended to filmy RA, the TRIP effect forms a localized plastic deformation zone, and crack extension is restrained by the increasing content of martensite [49]. This mechanism effectively enhances the strength of steel, and plasticity also remains due to the ideal combination of strength and plasticity. In Figure 14d, a combination of dimples and cleavage planes can be found, and flakes of tearing debris are observed, proving the high plasticity of Q&P370 W.

**Figure 15:**
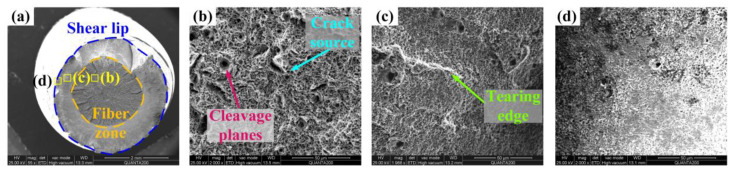
Tensile fracture of Q&P370 WU. (**a**) full view, (**b**) crack source in fiber zone, (**c**) tearing edge in shear lip, and (**d**) shear lip.

Then, the fracture morphology of Q&P370 WU is shown in Figure 15. Fluctuations in the fiber zone in Figure 15a are obvious, and cracks have a long extension process. Also, the edge between the fiber zone and the shear lip cuts off the crack. This morphology demonstrates the combination of strength and plasticity of steel. Significant plastic deformation in the core absorbs most of the energy, and the hardened layer hinders crack propagation. In Figure 15b, the crack source is surrounded by a large number of dimples. Larger dimples have cleavage planes in concentric circles, indicating that large dimples absorb more energy to postpone the fracture. Fewer dimples in Figure 15c are observed, especially in the area close to the hardened layer induced by the USR process. Most of the dimples have irregular rounded shapes, indicating that the area close to the hardened layer has lower plasticity. Figure 15d shows the area close to the surface, which shows very fine dimples mixed with a cleavage fracture, and the dimple size is significantly reduced compared to the core. This fracture morphology proves the effect of the USR process on strength enhancement, as severe plastic deformation notably refines the grain size and increases the surface hardness and strength. A harder surface leads to a core with higher plasticity, taking more energy and postponing the fracture, therefore exhibiting superior mechanical properties of the combination of strength and plasticity [22].

#### 3.3.3. Fracture of Q&P350 N and NU

**Figure 16:**
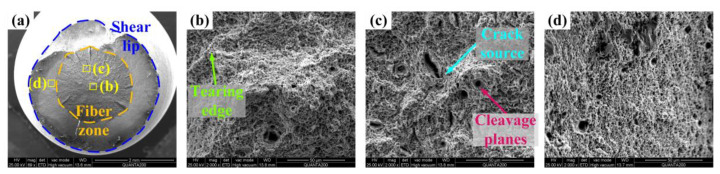
Tensile fracture of Q&P350 N. (**a**) full view, (**b**) tearing edge in fiber zone, (**c**) crack source and cleavage planes in fiber zone, and (**d**) shear lip.

The fiber zone of Q&P350 N in Figure 16a is fluctuant, and crack extension is intercepted by the shear lip. The dimensions of the core dimples in Figure 16b are consistent, indicating that the core has a limited amount of energy absorbed and the characteristic of brittleness. The crack source in Figure 16c is short and surrounded by dimples with notable size differences, and cleavage planes can be observed in limited numbers of large dimples. This fracture demonstrates that the fiber zone is brittle. The characteristic of quasi-cleavage can be observed in the shear lip in Figure 16d. The fracture morphology is consistent with the analysis above, in which a high content of unstable blocky RA with a coarse size could guarantee similar total elongation as Q&P370 W, but a high content of blocky RA and martensite also induces brittleness, leading to limited enhancement in tensile strength.

**Figure 17:**
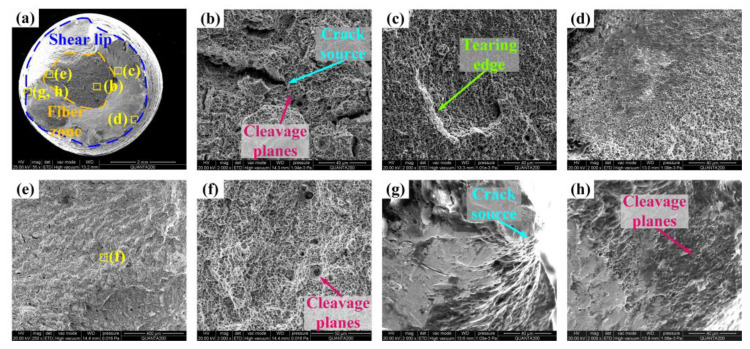
Tensile fracture of Q&P350 NU. (**a**) full view, (**b**) crack source and cleavage planes in fiber zone, (**c**) tearing edge in shear lip, (**d**) shear lip, (**e**) fiber zone, (**f**) cleavage planes in fiber zone, (**g**) crack source in shear lip, and (**h**) cleavage planes in shear lip.

Figure 17 shows the fracture morphology of Q&P350 NU. The fiber zone has no fluctuations, and the crack source is also observed in the shear lip in Figure 17a. The crack source in Figure 17b is surrounded by a large number of dimples with cleavage planes, and the dimples are uniform in size with irregular shapes. This morphology proves that more RA is transformed to martensite, and the fiber zone absorbs more energy than Q&P350 N with a harder surface. The shear lip in Figure 17c is composed of small dimples with tearing edges. The small dimple size indicates limited energy absorption and is macroscopically brittle. The section edge in Figure 17d also shows a combination of dimple and cleavage fractures, especially near the hardened layer. Those areas demonstrate the notable brittleness of the steel, as dimples that are limited in size cannot absorb much energy and cause rapid fracturing. This conclusion can also be demonstrated by Figure 17e, in which the fiber zone with less fluctuation indicates a fast fracture process. Also, only a limited amount of unstable blocky RA induces a few large dimples in Figure 17f, with many cleavage planes in concentric circles, which slow down the fracture extension. In addition, the further hardened surface causes a notable crack source in Figure 17g. The surface crack source with a large area of the cleavage plane in Figure 17h demonstrates the brittleness of steel treated by the USR process. In conclusion, a large amount of unstable blocky RA and martensite guarantees the brittleness and strength of steel, but with low plasticity and limited section shrinkage.

### 3.4. Mechanical Property Enhancement Mechanism

For Q&P370 W, high cooling speed in quenching generates bainite, and the formation of bainite splits the coarse components for high strength. Filmy RA induced by bainite with high stability guarantees the TRIP effect, which is a key factor in the high plasticity of steel. A slower cooling speed, such as in Q&P370 Oil and Q&P370 N, generates no bainite, but the gradient carbon distribution determines microstructural evolution. As the partitioning temperature is reduced, Q&P350 induces no bainite but rather a mild carbon redistribution process, and a slower cooling speed guarantees adequate carbon absorption. The component size was also refined in this process, leading to relatively stable blocky RA as seen in Q&P350 N, which guarantees high tensile strength and total elongation. The relatively stable blocky RA and coarse martensite induce a harder surface than AR, which also has an influence on the hardened layer depth in the USR process.

The USR process enhances the surface via high pressure, and the surface experiences severe plastic deformation with a significantly refined grain size. The hardened layer is also formed in this process, and RA is transformed into martensite. The effect of the USR process is influenced by the basic mechanical properties of steel. Specimens with high plasticity have a deeper hardened layer, such as Q&P370 WU, in which the hardened layer suppresses crack extension to promote the combination of strength and plasticity. This result proves the effect and feasibility of the combined process of Q&P and USR. In addition, a deeper hardened layer would induce a greater decline in plasticity, and the balance between the increase in strength and plasticity retention should be further studied to achieve better mechanical properties.

## 4. Conclusions

High-strength Cr-Ni-Mo martensitic steel has been greatly studied in surface strengthening, and its strength can be further improved with a core that has high plasticity, which can be induced by the Q&P process. With the induction of bainite, superior plasticity in the core could be guaranteed and the surface strength could be enhanced by the USR process to improve the mechanical properties of steel. In this paper, high-strength Cr-Ni-Mo martensitic steel is treated by the Q&P and USR processes to study the microstructure and mechanical property evolution. The tensile strength of specimens after the Q&P and USR processes has a two-fold increase compared to the AR specimen. The induction of bainite and the USR process guarantees the combination of strength of the surface and plasticity in the core. The conclusions from the above analysis are as follows:

(1) The Q&P process promotes microstructural evolution and carbon redistribution. Different partitioning temperatures with different cooling speeds induce variations in the microstructural composition and mechanical properties. With stable filmy RA, Q&P370 W has the best mechanical properties after the Q&P process, in which the volume fraction of RA for Q&P370 W is 10.81%, hardness is 437.76 HV_0.2_, tensile strength is 1389.76 MPa, total elongation is 11.34%, and PSE is 15.76 GPa%. Due to sufficient carbon absorption, Q&P350 N also shows a good performance, as the volume fraction of RA is 8.91%, hardness is 367.23 HV_0.2_, tensile strength is 1220.68 MPa, total elongation is 11.18%, and PSE is 13.65 GPa%. Compared to AR, Q&P370 W has an increase of 89.76% in hardness and 103.86% in tensile strength. Both Q&P370 W and Q&P350 N were further treated by the USR process in a surface-strengthening study.
(2) The USR process further enhances the mechanical properties and achieves a combination of superior strength and plasticity. Q&P370 W has a harder surface and high plasticity, and the USR process greatly improves the mechanical properties. For Q&P370 WU, the hardness is 487.63 HV_0.2_, the tensile strength improved to 1586.62 MPa, and PSE is 15.9 GPa%, while the total elongation is 10.02% and the RA volume is 2.96%. In comparison, Q&P350 N is hard and brittle, and the USR process only had a limited effect on mechanical property enhancement. Q&P350 NU showed values of 472.07 HV_0.2_, 1252.87 MPa, 10.22%, 12.8 GPa%, and 5.21% in hardness, tensile strength, total elongation, PSE, and RA volume, respectively. Compared to AR, Q&P370 WU has an increase of 111.38% in hardness and 132.73% in tensile strength. Specimens with high plasticity had deeper hardened layers, but the hardened layer depth also had a negative influence on total elongation. Q&P370 WU had a hardened surface with a core that was high in plasticity, which proves the combination of strength and plasticity after the combined process of Q&P and USR.

## Figures and Tables

**Table 1:** Compositions of Cr-Ni-Mo steel (wt.%).

Ni	Cr	Mn	Mo	C	Si	V	Al	Cu	N	Fe
2.1	1.55	0.66	0.3	0.26	0.26	0.11	0.1	0.03	0.02	Bal.

**Table 2:** The specimens tested and studied in this paper.

Specimen	Treatment
As-received (AR)	Spheroidal annealing, continuous rolling, and fine tuning of raw material
Q&P370 W	AR+ partitioning in 370 °C, 45 min, quenched by water
Q&P370 Oil	AR+ partitioning in 370 °C, 45 min, quenched by oil
Q&P370 N	AR+ partitioning in 370 °C, 45 min, quenched by normalizing
Q&P350 W	AR+ partitioning in 350 °C, 45 min, quenched by water
Q&P350 Oil	AR+ partitioning in 350 °C, 45 min, quenched by oil
Q&P350 N	AR+ partitioning in 350 °C, 45 min, quenched by normalizing
Q&P370 WU	Q&P370 W + USR process
Q&P350 NU	Q&P350 N + USR process

**Table 3:** The USR parameters for surface strengthening.

Feed υ	Static Force F	Amplitude A	Frequency ƒ	Palstance ω
0.063 mm/r	800 N	12 μm	25 kHz	35 rpm

**Table 4:** Carbon content of blocky RA in different specimens (wt.%).

Specimen	Q&P370 W	Q&P370 Oil	Q&P370 N	Q&P350 W	Q&P350 Oil	Q&P350 N
Carbon content	0.55	0.52	0.67	0.48	0.57	0.62

## Data Availability

The raw data supporting the conclusions of this article will be made available by the authors on request.

## Abbreviations

AR	As-received
B	Bainite
C	Carbide precipitate
CCT	Continuous cooling transformation
EDS	Energy dispersive spectrometer
FWHM	Full width at half maximum
$l_{1}$	Peak intensity of α(211) in XRD
$l_{2}$	Peak intensity of γ(220) in XRD
M	Martensite
M/A	Martensite/austenite
OM	Optical microscope
PSE	Product of strength and elongation
Q&P	Quenching and partitioning
Q&P370 W	AR+ partitioning in 370 °C, 45 min, quenched by water
Q&P370 Oil	AR+ partitioning in 370 °C, 45 min, quenched by oil
Q&P370 N	AR+ partitioning in 370 °C, 45 min, quenched by normalizing
Q&P350 W	AR+ partitioning in 350 °C, 45 min, quenched by water
Q&P350 Oil	AR+ partitioning in 350 °C, 45 min, quenched by oil
Q&P350 N	AR+ partitioning in 350 °C, 45 min, quenched by normalizing
Q&P370 WU	Q&P370 W + USR process
Q&P350 NU	Q&P350 N + USR process
RA	Retained austenite
SEM	Scanning electron microscope
SP	Stepped partitioning
TRIP	Transformation-induced plasticity
TTT	Time temperature transformation
USR	Ultrasonic surface rolling
$V_{RA}$	Volume fraction of RA
W	Water
XRD	X-ray diffraction
YS	Yield strength
